# Clinical Relevance and Methodological Comparison of Anti-MDA5 Antibody Detection: A Five-Year Retrospective and Exploratory Pilot Study

**DOI:** 10.3390/biom16050698

**Published:** 2026-05-08

**Authors:** Sándor Mogyoróssy, Gábor Nagy, Zoltán Griger, Melinda Nagy-Vincze, Monika Bodoki, Dóra Csige, Tünde Tarr, Éva Zöld, György Pfliegler, Boglárka Csilla Brúgós, Hui Lu, Sarah Tansley, Péter Antal-Szalmás, Andrea Domján, Szilvia Szamosi, Gabriella Szűcs, Zoltán Szekanecz, Ágnes Horváth, Levente Bodoki

**Affiliations:** 1 Department of Rheumatology, Faculty of Medicine, University of Debrecen, 4032 Debrecen, Hungary; sandormogyorossydr@gmail.com (S.M.); bodoki.monika@med.unideb.hu (M.B.); dora.csige96@gmail.com (D.C.); domjan.andrea@med.unideb.hu (A.D.); szamosi.szilvia@med.unideb.hu (S.S.); szucs.gabriella@med.unideb.hu (G.S.); szekanecz.zoltan@med.unideb.hu (Z.S.); kis.horvathagi@gmail.com (Á.H.); 2 Doctoral School of Medical Sciences, University of Debrecen, 4032 Debrecen, Hungary; 3 Department of Laboratory Medicine, Faculty of Medicine, University of Debrecen, 4032 Debrecen, Hungary; nagy.gabor@med.unideb.hu (G.N.); antalszp@med.unideb.hu (P.A.-S.); 4 Division of Clinical Immunology, Faculty of Medicine, University of Debrecen, 4032 Debrecen, Hungary; grigerz@googlemail.com (Z.G.); melinda.nagyvincze@gmail.com (M.N.-V.); tarr.tunde@med.unideb.hu (T.T.); zold_eva@yahoo.com (É.Z.); 5 Centre of Rare Diseases, Department of Internal Medicine, University of Debrecen, 4032 Debrecen, Hungary; g.pfliegler@gmail.com (G.P.); brugosb@gmail.com (B.C.B.); 6 Department of Life Sciences, University of Bath, Bath BA2 7AY, UK; hl225@bath.ac.uk (H.L.); slt32@bath.ac.uk (S.T.)

**Keywords:** anti-MDA5, dermatomyositis, idiopathic inflammatory myopathy, immunoblot, immunoprecipitation

## Abstract

Anti-melanoma differentiation-associated gene 5 (anti-MDA5) antibodies are critical biomarkers in myositis, associated with distinct clinical features and prognosis. This study aimed to evaluate the proportion of anti-MDA5 positivity and compare the diagnostic performance of local immunoblotting (IB) with gold-standard immunoprecipitation (IP). We performed a retrospective analysis of 3272 physician-requested anti-MDA5 IB determinations over a five-year period (2019–2023). A subsequent exploratory pilot study of ten Hungarian patients with myositis was conducted to compare IB results with radiolabeled protein IP. Confirmatory in-house enzyme-linked immunosorbent assay (ELISA) was used to distinguish between 140 kDa bands (anti-MDA5 vs. anti-NXP2). Indirect immunofluorescence (IIF) on HEp-2 cells was also evaluated. In the retrospective cohort, 3.7% (*n* = 121) of samples were non-negative. Among 64 borderline patients, only one (1.6%) had a definitive diagnosis of dermatomyositis (DM). Conversely, the proportion of confirmed myositis cases was notably higher among patients with strong positive IB results. In our exploratory cross-sectional pilot study, complete concordance between the two assays was observed for negative and strong positive results. Discrepancies were noted in borderline and weak positivity ranges, where anti-MDA5 was not detected by IP; instead, alternative autoantibodies were identified. The three IP-confirmed MDA5 positive samples were all validated by ELISA. The characteristic IIF cytoplasmic staining was identifiable in 2 out of 10 cases (20%). In our cohort, borderline IB cases were frequently potential false positives, highlighting the need for careful clinical evaluation. Borderline and weak results require clinical correlation or confirmatory testing to avoid misdiagnosis.

## 1. Introduction

Currently, myositis-specific antibodies (MSAs)/myositis-associated antibodies (MAAs) can be detected in around 60–70% of idiopathic inflammatory myopathy (IIM) patients. Anti-melanoma differentiation-associated gene 5 (anti-MDA5; also known as anti-CADM-140 or *IFIH1*) is an MSA that is routinely tested for and is associated with distinct clinical and prognostic features in patients with dermatomyositis (DM) and amyopathic dermatomyositis (ADM) [1,2,3,4,5,6,7]. Anti-MDA5 antibody was first identified by Sato et al. in 2005 in Japanese patients; its antigen is a 140 kDa cytoplasmic RNA-specific helicase encoded by the MDA5 gene [2,8,9,10,11]. The frequency of anti-MDA5 among myositis populations varies between 1% and 30% and is especially common in the Asian population [2,12,13]. Its clinical presentation is highly heterogeneous: in a study investigating EuroMyositis registry patients, anti-MDA5 antibody was only found in DM cases; other studies suggested that anti-MDA5 antibody may be the hallmark of ADM associated with rapidly progressive interstitial lung disease (RP-ILD) [7,14]. Anti-MDA5 positivity is generally associated with a poor prognosis and higher mortality [12,15,16,17,18]. From an immunological perspective, the MDA5 protein is an intracellular viral sensor that recognizes double-stranded RNA, playing a crucial role in the innate immune response and the subsequent activation of type I interferon pathways [12]. Antibody levels, typically measured by ELISA, correlate with disease activity, risk of relapse, and therapeutic response, making them valuable tools for clinical monitoring [12,16,17,19].

There are several methods that may be used for anti-MDA5 detection. Each has its own specific advantages and disadvantages/limitations, which should be considered when using a method. Immunoprecipitation (IP) is a useful method, and, according to the literature, it is considered to be the method of reference, the gold standard for the detection of myositis autoantibodies, including anti-MDA5 due to its high sensitivity and specificity [12,17,20,21]. IP is expensive, time-consuming, unable to provide antibody levels, and requires staff with expert, specific training as well as adequate facilities because of radioactivity in some forms [17,22,23,24]. Consequently, testing based on IP, or even ELISA utilizing in-house recombinant proteins, is not widely accessible for routine laboratory use [7]. As a result, commercial IB and ELISA kits have become increasingly popular, though they carry a risk of false-positive or false-negative results [12,17]. While IB offers a rapid, cost-effective multiplex approach providing semi-quantitative results, it may present certain specificity limitations [21,23,25]. Conversely, ELISA provides valuable quantitative data for disease monitoring, though broad MSA screening with this single-plex method can be less cost-effective [17,21].

The reliable detection of anti-MDA5 positivity is a critical challenge for both clinicians and laboratory analysts. For instance, assessing anti-MDA5 positivity is highly important for patients admitted to the intensive care unit with rapidly progressive interstitial lung disease (RP-ILD), because it may directly impact the applied therapy, often prompting the immediate initiation of aggressive, combined immunosuppressive regimens rather than standard step-up therapy due to the high mortality risk. Our primary objective was to evaluate the proportion of anti-MDA5 positivity and its clinical correlation in a large retrospective cohort from routine practice. Our secondary objective was to perform an exploratory pilot comparison of IB and IP agreement in a selected myositis cohort. Finally, as a tertiary objective, we explored the potential adjunctive role of antinuclear antibody (ANA) screening by HEp-2 IIF in resolving diagnostic discrepancies.

## 2. Materials and Methods

### 2.1. Study Design and Patient Selection

This study employed a two-phase design. In the retrospective phase of the study, data were retrieved from the laboratory information system of the Department of Laboratory Medicine at the University of Debrecen for a five-year period between 1 January 2019 and 31 December 2023. All physician-requested determinations for anti-MDA5 detection by IB were analyzed. We acknowledge that this cohort is subject to selection bias, as testing was driven by clinical suspicion rather than systematic screening of a defined IIM population. From this database, we identified patients whose samples showed at least weak anti-MDA5 positivity by IB. Additionally, one patient (Patient 3) with a persistent borderline result but a definitive DM diagnosis was included to explore the clinical relevance of borderline signals. The explicit inclusion criteria for the pilot study were: (1) a definitive diagnosis of IIM, and (2) at least one non-negative anti-MDA5 IB result. Patients lacking a definitive myositis diagnosis, those lost to follow-up, or those with other overlapping systemic autoimmune diseases without definitive myositis features were excluded, as outlined in the flowchart.

**Figure 1:**
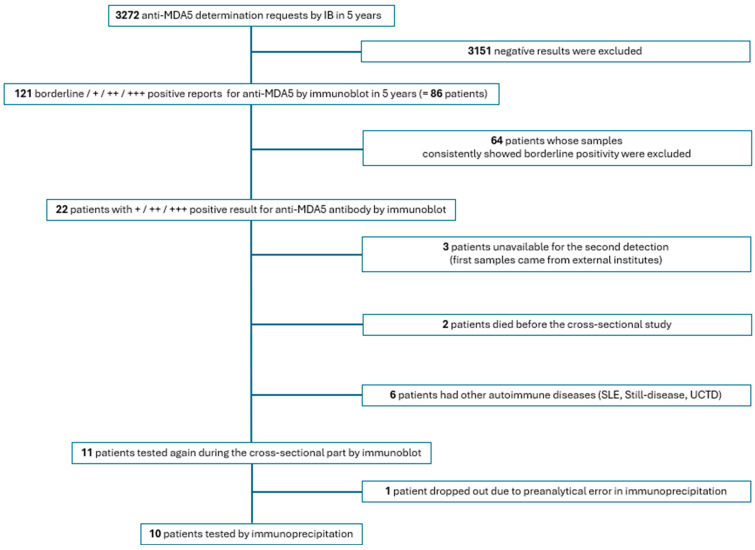
Flowchart of the study. Abbreviations: MDA5: melanoma differentiation-associated protein 5, SLE: systemic lupus erythematosus, UCTD: undifferentiated connective tissue disease, +: weak positive, ++: moderate positive, +++: strong positive.

For the subsequent pilot cross-sectional phase, 11 patients with myositis were initially enrolled from the Department of Rheumatology and Immunology and the Division of Clinical Immunology at the Faculty of Medicine, University of Debrecen. The selection process is detailed in Figure 1.

All included patients had a definitive diagnosis of myositis according to the Bohan and Peter diagnostic criteria or the ACR/EULAR classification criteria for IIMs. Furthermore, each patient had demonstrated anti-MDA5 positivity by the local laboratory’s IB method at least once following their diagnosis. Medical records were reviewed to collect clinical data for population description and statistical analysis.

### 2.2. Sample Collection and Local Immunoblot Testing

Peripheral blood samples were collected simultaneously from all 11 patients by trained healthcare professionals. These samples were used to repeat the local IB testing, while serum aliquots were stored at −80 °C and subsequently shipped to the United Kingdom for IP analysis. It is important to note that sample 11 had to be excluded from the subsequent IP analysis due to a pre-analytical error during sample processing. Consequently, the IP comparison was performed on a final cohort of 10 samples.

The local laboratory utilized the Autoimmune Inflammatory Myopathies 16 Ag immunoblot (technically a line blot) reagent kit (cat. No.: DL 1530-1601-4 G) from Euroimmun AG (Lübeck, Germany) for routine anti-MDA5 detection. During the test, 16 autoantibodies were determined simultaneously—autoantibodies against Mi-2α, Mi-2β, TIF1γ, MDA5, NXP2, SAE1, Ku, PM/Scl100, PM/Scl75, Jo-1, SRP, PL-7, PL-12, EJ, OJ, Ro-52. Blood samples were forwarded to our local laboratory immediately after collection. The kit was used according to the manufacturer’s instructions. Serum samples were diluted in a 1:101 ratio. Test strips were scanned and evaluated in the EuroLineScan software (version 3.4.37). Colour intensity of each antibody band was determined as a numeric value and reported on a semi-quantitative scale (negative “−”, borderline “+/−“, weak positive “+”, moderate positive “++”, strong positive “+++”), categorized using the default manufacturer thresholds (signal intensity < 5: negative; 6–10: borderline; 11–25: weak positive; 26–50: moderate positive; >50: strong positive).

### 2.3. Immunoprecipitation and Confirmatory Assays

Anti-MDA5 antibodies were also detected by radiolabeled protein immunoprecipitation in an independent laboratory at the University of Bath, Bath, UK. Serum aliquots of 200 µL were shipped on dry ice in accordance with health regulations. Briefly, 10 μL sera was mixed with 2 mg protein-A-Sepharose beads (Sigma, Gillingham, UK) in IPP buffer (10 mM Tris–Cl pH 8.0, 500 mM NaCl, 0.1% *v*/*v* Igepal) at room temperature for 30 min. Beads were washed in IPP buffer prior to the addition of 120 μL [35S] methionine labelled K562 cell extract. Samples were mixed at 4 °C for 2 h. Beads were washed in IPP buffer followed by TBS buffer (10 mM Tris–Cl pH 7.4, 150 mM NaCl) before being resuspended in 50 μL SDS sample buffer (Sigma, Gillingham, UK). After heating, proteins were fractionated by 10% SDS PAGE, enhanced, fixed and dried. Labelled proteins were analyzed by autoradiography.

Since both anti-MDA5 and anti-NXP2 produce a band at 140 kDa on IP, in-house anti-MDA5 and anti-NXP2 ELISAs developed in Bath were utilized to confirm the specific identity of the bands. These confirmatory measurements were also performed at the Bath facility [14].

### 2.4. Indirect Immunofluorescence

Nuclear, cytoplasmic and mitotic antinuclear antibody (ANA) patterns were detected on HEp-2 cells using an automated indirect immunofluorescence assay (cat. No.: FC 1522-1010, Euroimmun AG, Lübeck, Germany). Screening dilution was 1:80, positive samples were titrated up to 1:5120. The IIF slides were evaluated by two experienced clinical laboratory specialists who were blinded to the patients’ IB and IP results. While the automated Euroimmun software (version 3.4.37) provided initial pattern recognition, all final readings and pattern interpretations were confirmed by mandatory expert microscopic review.

### 2.5. Statistical Analysis

Statistical analysis was performed using IBM SPSS Statistics version 27.0 (IBM Corp., Armonk, NY, USA) to describe the demographic and clinical characteristics of the cohort. Descriptive statistics were utilized to summarize the data, with categorical variables reported as absolute frequencies and percentages. To evaluate the diagnostic agreement between the local immunoblot assay and the gold-standard immunoprecipitation technique in our exploratory cohort, overall percentage agreement and Cohen’s kappa (κ) were calculated. Furthermore, positive predictive value (PPV) and negative predictive value (NPV) were estimated. For all proportion metrics, 95% confidence intervals (95% CI) were calculated using the Wilson score method. Given the small sample size of this pilot cohort, these statistics are strictly exploratory, and advanced correlational analyses were not pursued to avoid underpowered conclusions.

### 2.6. Ethical Approval

The study was approved by the Hungarian Scientific Research Council Ethical Committee (approval No. BM/11321-3/2023/EKU). Written informed consent was obtained from each patient, and the study was carried out according to the Declaration of Helsinki and its amendments.

## 3. Results

### 3.1. Retrospective Analysis of Anti-MDA5 Positivity Rate and Clinical Correlation

Over the five-year study period, the laboratory performed 3272 anti-MDA5 determinations. The results were negative in 3151 samples (96.3%), while the results were “not negative” in 121 samples (3.7%). These non-negative cases were reported by IB as 84 borderline (2.57%), 16 weak positive (0.49%), 5 moderate positive (0.15%), and 16 strong positive (0.49%) anti-MDA5 results. These 121 samples were taken from 86 patients due to repetitive testing in some cases; 22 of these patients had weak, moderate, or strong positivity, while 64 patients had borderline positivity.

Out of the 64 borderline positive patients (representing 84 borderline results), only one had DM (1.56%), whereas myositis could not be confirmed in the remaining 63 patients (98.44%). The 16 weak positive results originated from 14 patients: 7 patients (50%) had a definitive diagnosis of myositis (4 DM, 2 PM and 1 ADM); 6 patients had other autoimmune diseases (polymyalgia rheumatica, undifferentiated connective tissue disease (UCTD), systemic lupus erythematosus or Still’s disease), and one sample came from an external institution with unavailable clinical data. The 5 moderate positive results came from 3 patients, of whom 2 had a definitive diagnosis of DM (66.67%) and one had UCTD. The 16 strong positive results originated from 9 patients: 6 patients had a definitive diagnosis of DM (66.67%), 1 patient had UCTD, and two patient samples came from an external institution without available clinical data. Summing the weak, moderate, and strong (+/++/+++) categories yields 26 instances, which actually represent 22 unique patients, as the immunoblot intensities of some patients fluctuated during follow-up. Based on the principles shown in the flowchart (Figure 1), ten patients were selected for further investigations, and their most important clinical characteristics are shown in Table 1.

### 3.2. Pilot Cross-Sectional Comparison: Immunoblot vs. Immunoprecipitation

Although 11 patients were initially enrolled and evaluated by the local immunoblot method, one patient’s sample was excluded due to a pre-analytical error prior to IP testing, as detailed in the Methods. Therefore, to ensure consistency across all comparative analyses, the tables and the results below present the final standardized cohort of 10 patients. At the time of diagnosis, the initial detection of anti-MDA5 antibodies yielded the following results: five patients exhibited strong positivity (samples 2, 5, 7, 9, and 10), one showed moderate positivity (sample 6), four had weak positivity (samples 1, 4, 8, and 11), and one displayed a borderline result (sample 3) (Table 2).

Upon re-testing during the current study, three patients remained strongly positive (samples 2, 5, and 7), one was moderately positive (sample 6), two were weakly positive (samples 4 and 11), one showed a borderline result (sample 9), and four tested negative (samples 1, 3, 8, and 10) (Table 2 and Table 3).

Based on the IP findings, this 10-patient cohort could be stratified into four distinct groups. IP confirmed anti-MDA5 positivity in only 3 of the 10 samples (samples 2, 5, and 7); notably, all three also exhibited additional unidentified bands. Among the anti-MDA5 negative samples, alternative autoantibodies were detected: samples 4 and 9 were positive for anti-RuvBL1/2 and anti-U1RNP, respectively. Furthermore, samples 6 and 10 displayed unidentified bands that did not correspond to any known autoantibodies. Finally, samples 1, 3, and 8 were entirely negative by IP (Table 3 and Table 5).

During confirmatory testing, the three IP-positive samples for MDA5 (2, 5, and 7) were uniformly positive on the anti-MDA5 ELISA and negative on the anti-NXP2 ELISA.

To summarize the diagnostic agreement between the local immunoblot assay and the gold-standard immunoprecipitation within our exploratory cohort, a contingency table was constructed (Table 4).

Despite the small sample size, we calculated exploratory predictive values for the IB method based on the re-testing data: when considering borderline cases as positive, the positive predictive value was 50.0% (95% CI: 18.8–81.2%), while the negative predictive value was 100.0% (95% CI: 51.0–100.0%). The overall percentage agreement between the two methods was 70.0% (95% CI: 39.7–89.2%), with a Cohen’s kappa coefficient of 0.44, indicating moderate agreement. However, no formal validation of diagnostic performance can be inferred from this exploratory pilot cohort.

Descriptively comparing the clinical phenotypes, all three IP-confirmed anti-MDA5 positive patients (Patients 2, 5, 7) presented with classic dermatomyositis diagnoses, whereas the IB-positive/IP-negative group was clinically more heterogeneous, consisting of both dermatomyositis and polymyositis cases. Regarding the two patients labeled as polymyositis (Patients 4 and 8), the diagnoses were based on the Bohan and Peter criteria. Patient 4 had a definitive diagnosis confirmed by muscle biopsy and electromyography in 2005. Both patients have been regularly followed at our clinical center for 19 and 25 years, respectively, and during this extensive longitudinal follow-up, no overlap features or other connective tissue disease-specific antibodies were identified, confirming the diagnosis of isolated myositis.

### 3.3. ANA Pattern Analysis

Nuclear, cytoplasmic, and mitotic ANA patterns were detected on HEp-2 cells. Three samples were ANA negative, and 6/10 patients were positive for nuclear ANA (Table 5).

**Figure 2:**
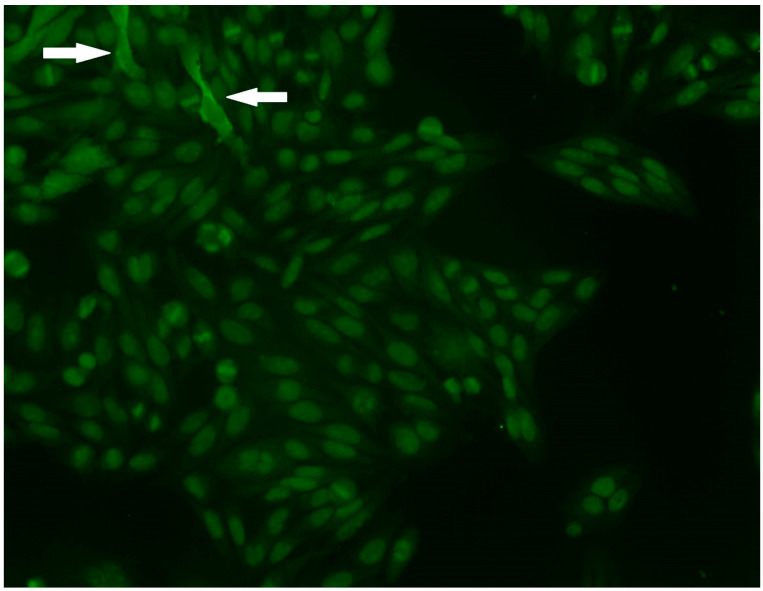
This is the picture of the HEp-2 indirect immunofluorescence assay of patient 5 at a dilution of 1:80. The cytoplasmic pattern that is said to be relatively characteristic of MDA5 can be seen as dense fine speckled cytoplasmic staining in two cells (white arrows).

Only two samples exhibited the characteristic IIF pattern associated with anti-MDA5 antibodies, which typically presents as a fine granular or speckled fluorescence distributed throughout the cytoplasm, reflecting the intracellular localization of the MDA5 viral sensor (Figure 2).

Discrepancies between serological and immunofluorescence results were a notable feature of our cohort. For instance, although Patient 6 demonstrated the characteristic fine speckled cytoplasmic pattern on HEp-2 cells, the gold-standard IP assay yielded a negative result for anti-MDA5. Conversely, Patient 7, who had strong anti-MDA5 positivity confirmed by both IB and IP, showed no characteristic cytoplasmic staining. These contradictory findings further highlight the diagnostic limitations of relying on routine IIF screening for anti-MDA5 detection.

## 4. Discussion

In the retrospective phase of our study, we observed that among 64 borderline positive cases, only one patient had a definitive diagnosis of myositis. This finding suggests that a borderline (+/−) immunoblot result is frequently a potential false positive in our specific clinical context, and definitive IIM rarely occurs in such cases. Conversely, as the intensity of the immunoblot signal increased from weak (+) to strong (+++), the frequency of definitive myositis diagnoses also rose, reaching 50% and 66.67%, respectively. These results indicate that, from a clinical perspective, interpreting low-intensity signals requires extreme caution, and future large-scale studies might consider evaluating optimal cut-off values to increase the specificity of the test.

It is important to note that even among strong positive cases, a definitive myositis diagnosis was not universal. In two such cases, clinical data were unavailable, though it is assumed these patients, treated in other regions of Hungary, were also diagnosed with DM. Furthermore, the appearance of other autoimmune diseases in our cohort suggests that the anti-MDA5 antibody may not be as specific for myositis as previously hypothesized. Our review of clinical records confirmed that these were not overlap syndromes, as the patients lacked typical muscle weakness or DM-like skin symptoms. Although monitoring UCTD cases for potential progression to IIM is clinically justified, our cross-sectional study focused exclusively on definitive myositis patients. This focus explains the inclusion of Patient 3, who initially presented with a borderline result but had a definitive DM diagnosis. Interestingly, this patient’s antibody positivity fluctuated over time before eventually disappearing during the study period. Furthermore, two patients initially identified with PM and weak IB positivity were later found to be anti-MDA5 negative by IP, suggesting they were likely discordant, potential false positives. This reinforces the clinical observation that ‘true’ anti-MDA5 positive patients in our study always presented with characteristic skin symptoms.

The disappearance of the antibody in five patients during the cross-sectional study can be attributed to the well-known phenomenon where anti-MDA5 levels decrease or become undetectable following appropriate immunosuppressive treatment. As Gono et al. noted, managing false-positive and false-negative results remains a significant challenge when utilizing commercial MSA assays [26].

While various research groups have employed commercially available IB kits [27,28,29,30,31,32,33] or IP and ELISA techniques [3,6,7,13,17,34,35,36,37] for anti-MDA5 detection, our pilot study aimed to validate our local IB method against the gold-standard IP technique in an independent laboratory. Our comparative analysis showed agreement in the majority of cases (7 out of 10 samples), specifically for Patients 1, 2, 3, 5, 7, 8, and 10. Discrepancies were observed in Patients 4, 6, and 9, where IB indicated some level of positivity that IP could not confirm. Overall, negative and strong positive (+++) IB results were in complete agreement with IP. The lack of confirmation for borderline and weak IB results by IP is most likely attributable to the lower specificity and potential cross-reactivity of the immunoblot method, although the detection limits of the immunoprecipitation technique cannot be entirely ruled out.

Alternative explanations for these discordant IB+/IP- results must also be thoroughly considered. Commercial immunoblotting utilizes denatured, linear recombinant antigens, which may expose hidden epitopes that are inaccessible in the folded, native proteins used in immunoprecipitation. Furthermore, while IP is considered the gold standard, it might lack the sensitivity to detect very low-titer antibodies, or the antibodies might have lost their affinity due to preanalytical degradation. Finally, as our pilot study focused explicitly on the clinical dilemma of resolving non-negative (borderline/weak) IB results, the exclusion of IB-negative samples inherently prevents the calculation of true sensitivity, specificity, and false-negative rates. We acknowledge this as a limitation of our exploratory design.

We must emphasize that these metrics are derived from a highly selected, small pilot cohort (*n* = 10); therefore, they reflect the performance within this specific exploratory setting rather than generalizable diagnostic accuracy.

The IP method offers the advantage of detecting unknown antibodies or those not included in the IB panel, as demonstrated by the detection of anti-RuvBL1/2 in Patient 4. However, even IP (using K562 cell lysate) has limitations, such as the failure to detect the 52 kDa band in anti-Ro52 positive samples. Similar methodological comparisons have been explored in the literature; for instance, a study suggested that combining IP with IB or commercial ELISAs could serve as a viable alternative to IP alone [38]. The frequency of anti-MDA5 antibodies exhibits well-documented ethnic and geographic disparities, being notably more prevalent in Asian populations compared to European cohorts [1,14]. Interestingly, a previous study of a Hungarian IIM cohort of 337 patients using the same IP method failed to identify any anti-MDA5 positive samples, which perfectly aligns with our findings and confirms that this autoantibody is particularly rare in the Hungarian myositis population [39]. Other studies, such as those by Angeli et al. (2024) and Cavazzana et al. (2016) in Italian cohorts, have reported varying levels of agreement between line blot and IP methods [23,40].

Regarding indirect immunofluorescence, although most MSA/MAAs have corresponding HEp-2 staining patterns, the method is limited by low sensitivity and specificity, and a negative IIF result does not preclude the presence of an MSA [41]. In our study, the characteristic fine speckled cytoplasmic pattern of anti-MDA5 was identifiable in only a few cases, suggesting that in this small cohort, HEp-2 IIF showed limited sensitivity and inconsistent correlation with anti-MDA5 status.

One of the main strengths of the present study is that comprehensive clinical data were available and analyzed for the vast majority of our cohort. Finally, our study has certain limitations. The retrospective cohort consisted of physician-requested tests, introducing inherent selection and spectrum bias, which may overestimate apparent agreement in high-positive samples while underrepresenting clinically suspected IB-negative cases. Additionally, the small sample size—stemming from the rarity of this antibody in Hungary and the inclusion of only two centers—limits the generalizability of our prevalence estimates and precludes adequately powered correlation analyses. Lastly, as per standard real-world clinical laboratory protocols, the diagnostic assays were performed in singlicate rather than technical replicates, which we acknowledge as a minor methodological limitation.

## 5. Conclusions

One of the primary messages of our study is that antibody testing results in myositis must always be interpreted within the specific clinical context. Regarding the detection of anti-MDA5 via IB, we conclude that borderline cases should be interpreted with high clinical suspicion and correlated closely with the patient’s presentation, as they frequently did not align with IP confirmation in our cohort. In our comparative analysis, the IB and IP methods demonstrated agreement in the majority of cases. The concordant negative results observed between the two assays may be explained by the well-documented correlation between anti-MDA5 antibody levels, disease activity, and response to treatment. However, discrepancies were also observed in a subset of cases, particularly where IB indicated borderline or moderate positivity while IP yielded negative results. Given these findings, we suggest that in ambiguous clinical situations, repeat testing is essential to draw definitive conclusions.

Therefore, the development of standardized recommendations regarding the reliability of myositis autoantibody assays is urgently needed. To our knowledge, this is the first study to investigate a Hungarian IIM cohort by simultaneously employing different detection methods for the anti-MDA5 antibody. This exploratory work provides a crucial foundation for future, larger multicenter studies to comprehensively map the anti-MDA5-positive population in Hungary.

## Figures and Tables

**Table 1:** Clinical characteristics and phenotypes of the anti-MDA5-positive pilot cohort (*n* = 10).

Patient No.	Age at 2nd Sample/Sex	Diagnosis (Year)	ILD/ RP-ILD	Skin Symptoms	Muscle Involvement	Therapy at 2nd Sample
1	48/M	DM (2019)	Alveolitis, Fibrosis	Gottron’s papule, Heliotrop rash, Mechanic’s hand	Hypomyopathic	None (Remission)
2	58/F	DM (2020)	None	Periorbital edema	Mild weakness	None
3	67/M	DM (2008)	None	Heliotrop rash, Gottron’s sign, V-sign	Weakness, Myalgia	Glucocorticoid
4	57/F	PM (2005)	Fibrosis	None	Weakness, Myalgia	Glucocorticoid
5	48/F	DM (2022)	None	Heliotrop rash, Gottron’s sign, Subcutaneous calcinosis	Weakness	GC + Methotrexate
6	66/F	DM (2013)	Alveolitis	Periorbital edema	Weakness	None
7	55/F	DM (2020)	Alveolitis, Fibrosis	Mechanic’s hand, Gottron’s sign	Weakness	GC + Cyclosporin A
8	63/F	PM (1998)	None	None	Weakness, Myalgia	Glucocorticoid
9	61/F	DM (2020)	Alveolitis, Fibrosis	Erythema, Mechanic’s hand	Weakness, Atrophy	Mycophenolate mofetil
10	47/F	DM (2020)	Alveolitis, Fibrosis	Gottron’s papule, Edema, V-sign	Weakness	GC + Methotrexate

Abbreviations: DM: dermatomyositis, F: female, GC: glucocorticoid, ILD: interstitial lung disease, M: male, PM: polymyositis, RP-ILD: rapidly progressive interstitial lung disease.

**Table 2:** Results of anti-MDA5 antibody detection, using immunoblot.

Patient	First Detection (at the Diagnosis of the Myositis)	Second Detection (During the Study)
1	+	−
2	+++	+++
3	+/−	−
4	+	+
5	+++	+++
6	++	++
7	+++	+++
8	+	−
9	+++	+/−
10	+++	−

−: negative, +/−: borderline, +: weak positive, ++: moderate positive, +++: strong positive.

**Table 3:** Comparison of anti-MDA5 detection by immunoblot (IB) and immunoprecipitation (IP).

Patient	IB Anti-MDA5 Category	IB Anti-MDA5 Color Intensity (Ref.: <5)	IP Anti-MDA5	Concordance	Time Interval Between Samples (Years)
1	Negative	2	Negative	Yes	4
2	+++	57	Positive	Yes	3
3	Negative	5	Negative	Yes	4
4	+	16	Negative	No	4
5	+++	92	Positive	Yes	1
6	++	26	Negative	No	4
7	+++	67	Positive	Yes	1
8	Negative	3	Negative	Yes	4
9	+/−	7	Negative	No	3
10	Negative	4	Negative	Yes	4

Abbreviations: IB: immunoblot, IP: immunoprecipitation, MDA5: melanoma differentiation-associated gene 5, +/−: borderline, +: weak positive, ++: moderate positive, +++: strong positive.

**Table 4:** Diagnostic agreement between Immunoblot and Immunoprecipitation.

	IP Positive	IP Negative	Total
IB Positive *	3	3	6
IB Negative	0	4	4
Total	3	7	10

* IB Positive includes borderline, weak, moderate, and strong results based on the initial assay cut-offs. Abbreviations: IB: immunoblot, IP: immunoprecipitation.

**Table 5:** Additional autoantibody profiles and HEp-2 indirect immunofluorescence (IIF) patterns.

Patient	IB Other Antibodies	IP Other Antibodies	HEp-2 IIF Nuclear Pattern/Titer	HEp-2 IIF Cytoplasmic Pattern Corresponding to Anti-MDA5
1	all negative		nucleolar 1:80 titer	negative
2	Ro-52 +++, NXP2 +/−	unknown 32 kDa and 34 kDa bands *	negative	negative
3	all negative		nuclear homogeneous 1:320 and nucleolar 1:320 titer	negative
4	PM/Scl-75 +	anti-RuvBL1/2	nuclear speckled 1:640 titer	negative
5	PL-7 +/−, PM/Scl-100 +/−	unknown 24 kDa, 23 kDa and 21 kDa bands *	negative	positive
6	Ku +/−	unknown 54 kDa band *	nuclear homogeneous 1:80 and nuclear speckled 1:80 titer	positive
7	Ro-52 +++	unknown 56 kDa band *	nuclear speckled 1:640 and nuclear homogeneous 1:320 titer	negative
8	all negative		negative	negative
9	Ro-52 +++	anti-U1 and unknown 139 kDa and 160 kDa bands *	negative	negative
10	Ro-52 +++	unknown 63 kDa band *	nuclear homogeneous 1:160 titer	negative

* Unidentified bands that do not correspond to currently known myositis autoantibodies. Abbreviations: IB: immunoblot, IIF: Indirect immunofluorescence, IP: immunoprecipitation, MDA5: melanoma differentiation-associated gene 5, +/−: borderline, +++: strong positive.

## Data Availability

The data presented in this study are available on request from the corresponding author.

## Abbreviations

The following abbreviations are used in this manuscript:

ACR	American College of Rheumatology
ADM	Amyopathic dermatomyositis
AMA	American Medical Association
ANA	Antinuclear antibody
Anti-MDA5	Anti-melanoma differentiation-associated gene 5
CADM	Clinically amyopathic dermatomyositis
DM	Dermatomyositis
ELISA	Enzyme-linked immunosorbent assay
EULAR	European Alliance of Associations for Rheumatology
HEp-2	Human epithelioma type 2 (cells)
IB	Immunoblot
*IFIH1*	Interferon induced with helicase C domain 1
IIF	Indirect immunofluorescence
IIM	Idiopathic inflammatory myopathy
IP	Immunoprecipitation
MAAs	Myositis-associated antibodies
MSAs	Myositis-specific antibodies
NPV	Negative predictive value
PM	Polymyositis
PPV	Positive predictive value
RP-ILD	Rapidly progressive interstitial lung disease
UCTD	Undifferentiated connective tissue disease
